# Supplementary material for: Structural basis of stepwise G protein activation by the viral chemokine receptor US28
Source: Nat Commun. 2026 Sep 28;17:11171. doi: 10.1038/s41467-026-78122-7 (PMC13714248; doi:10.1038/s41467-026-78122-7)
Supplement: Supplementary file 2 [file 41467_2026_78122_MOESM2_ESM.pdf]

nature portfolio

Corresponding author(s): Naotaka Tsutsumi

Last updated by author(s): Aug 7, 2026

Reporting Summary

Nature Portfolio wishes to improve the reproducibility of the work that we publish. This form provides structure for consistency and transparency in reporting. For further information on Nature Portfolio policies, see our Editorial Policies and the Editorial Policy Checklist.

Statistics

For all statistical analyses, confirm that the following items are present in the figure legend, table legend, main text, or Methods section.

n/a	Confirmed	
☐	☒	The exact sample size (*n*) for each experimental group/condition, given as a discrete number and unit of measurement
☐	☒	A statement on whether measurements were taken from distinct samples or whether the same sample was measured repeatedly
☐	☒	The statistical test(s) used AND whether they are one- or two-sided<br>*Only common tests should be described solely by name; describe more complex techniques in the Methods section.*
☒	☐	A description of all covariates tested
☐	☒	A description of any assumptions or corrections, such as tests of normality and adjustment for multiple comparisons
☐	☒	A full description of the statistical parameters including central tendency (e.g. means) or other basic estimates (e.g. regression coefficient) AND variation (e.g. standard deviation) or associated estimates of uncertainty (e.g. confidence intervals)
☐	☒	For null hypothesis testing, the test statistic (e.g. *F*, *t*, *r*) with confidence intervals, effect sizes, degrees of freedom and *P* value noted<br>*Give P values as exact values whenever suitable.*
☒	☐	For Bayesian analysis, information on the choice of priors and Markov chain Monte Carlo settings
☒	☐	For hierarchical and complex designs, identification of the appropriate level for tests and full reporting of outcomes
☒	☐	Estimates of effect sizes (e.g. Cohen's *d*, Pearson's *r*), indicating how they were calculated

Our web collection on statistics for biologists contains articles on many of the points above.

Software and code

Policy information about availability of computer code

Data collection	SerialEM (version 3), Gatan DigitalMicrograph, PPM 3.0 Web Server, CHARMM-GUI, OpenMM (version 8.3.1)
Data analysis	cryoSPARC (version 4), Topaz (version 0.2.5a), UCSF ChimeraX (version 1.9), AlphaFold3, Coot (version 0.9.8.96), PHENIX (version 1.21.1_5286), PyMOL (version 3.1), GraphPad Prism 10, FlowJo, catdcd (version 4.0b), MDTraj (version 1.11.0), VMD (version 1.9.4a57)

For manuscripts utilizing custom algorithms or software that are central to the research but not yet described in published literature, software must be made available to editors and reviewers. We strongly encourage code deposition in a community repository (e.g. GitHub). See the Nature Portfolio guidelines for submitting code & software for further information.

Data

Policy information about availability of data

All manuscripts must include a data availability statement. This statement should provide the following information, where applicable:

- Accession codes, unique identifiers, or web links for publicly available datasets
- A description of any restrictions on data availability
- For clinical datasets or third party data, please ensure that the statement adheres to our policy

The cryo-EM maps and model coordinates have been deposited in the Electron Microscopy Data Bank (EMDB) and the Protein Data Bank (PDB) under accession codes EMD-66864/66942 (PDB: 9XH2/9XJP), EMD-66923 (PDB: 9XIY), EMD-66922/66928 (PDB: 9XIX/9XJ5), and EMD-66930 (PDB: 9XJ7) for the E-state, T2C-state, C-

state, and C'-state complexes, respectively. Curated raw cryo-EM movies have been deposited in the Electron Microscopy Public Image Archive (EMPIAR) under accession code EMPIAR-13238. Simulation data have been deposited in a Zenodo repository under entry 20598927. Source data are provided with this paper.

Research involving human participants, their data, or biological material

Policy information about studies with human participants or human data. See also policy information about sex, gender (identity/presentation), and sexual orientation and race, ethnicity and racism.

Reporting on sex and gender	N/A
Reporting on race, ethnicity, or other socially relevant groupings	N/A
Population characteristics	N/A
Recruitment	N/A
Ethics oversight	N/A

Note that full information on the approval of the study protocol must also be provided in the manuscript.

Field-specific reporting

Please select the one below that is the best fit for your research. If you are not sure, read the appropriate sections before making your selection.

☒ Life sciences ☐ Behavioural & social sciences ☐ Ecological, evolutionary & environmental sciences

For a reference copy of the document with all sections, see nature.com/documents/nr-reporting-summary-flat.pdf

Life sciences study design

All studies must disclose on these points even when the disclosure is negative.

Sample size	No statistical methods were used to pre-determine the sample size. Sample sizes for the cell-based assays (n ≥ 3) were chosen based on standard practices in the field to ensure reproducibility.
Data exclusions	No data were excluded from the final analyses. For cryo-EM data processing, low-quality micrographs and particles were filtered out using standard classification methods, as detailed in the Methods section.
Replication	Cell-based experiments were independently performed at least three times. All attempts at replication were successful.
Randomization	Randomization was not applicable to this study, as it relies strictly on highly controlled in vitro structural and biochemical assays where experimental conditions are uniform and lack heterogeneous populations requiring bias mitigation.
Blinding	No blinding was attempted or needed.

Reporting for specific materials, systems and methods

We require information from authors about some types of materials, experimental systems and methods used in many studies. Here, indicate whether each material, system or method listed is relevant to your study. If you are not sure if a list item applies to your research, read the appropriate section before selecting a response.

Materials & experimental systems

n/a	Involved in the study	
☐	☒	Antibodies
☐	☒	Eukaryotic cell lines
☒	☐	Palaeontology and archaeology
☒	☐	Animals and other organisms
☒	☐	Clinical data
☒	☐	Dual use research of concern
☒	☐	Plants

Methods

n/a	Involved in the study	
☒	☐	ChIP-seq
☐	☒	Flow cytometry
☒	☐	MRI-based neuroimaging

Antibodies

Antibodies used	An in-house anti-FLAG M1 antibody was used to prepare the affinity resin for protein purification.<br>Mouse anti-FLAG epitope (DYKDDDDK) tag monoclonal antibody (Clone 1E6, FujiFilm Wako Pure Chemicals, used at 10 µg/mL) was used as the primary antibody for flow cytometry.<br>Goat anti-mouse IgG secondary antibody conjugated with Alexa Fluor 488 (Thermo Fisher Scientific, used at 10 µg/mL) was used for flow cytometry.
Validation	The in-house anti-FLAG M1 antibody was validated by the successful affinity purification of the target GPCR complex, as confirmed by SDS-PAGE and size-exclusion chromatography.<br>The commercial primary and secondary antibodies were validated for flow cytometry by their respective manufacturers.

Eukaryotic cell lines

Policy information about cell lines and Sex and Gender in Research

Cell line source(s)	Spodoptera frugiperda (Sf9) cells: Used for insect baculovirus and BacMam virus preparation (ATCC, CRL-1711; RRID:CVCL_0549).<br>Trichoplusia ni (High Five) cells: Used for scFv16 production (Expression Systems, Cat# 94-002).<br>HEK293S GnTI⁻ cells: Used for BacMam protein production (ATCC, CRL-3022; gift from Prof. H. Gobind Khorana).<br>M1 hybridoma cells: Used to prepare the FLAG affinity resin (gift from Prof. Brian K. Kobilka).<br>HEK293A cells: Used for functional assays (Thermo Fisher Scientific, Cat# R70507).
Authentication	Cell lines were authenticated by the supplier. No additional authentication was performed by the authors in this study.
Mycoplasma contamination	Mycoplasma contamination was routinely assessed using the MycoAlert Mycoplasma Detection Kit (Lonza).
Commonly misidentified lines (See ICLAC register)	No cell lines used in this study are listed as commonly misidentified.

Plants

Seed stocks	N/A
Novel plant genotypes	N/A
Authentication	N/A

Flow Cytometry

Plots

Confirm that:

- [ ] The axis labels state the marker and fluorochrome used (e.g. CD4-FITC).
- [ ] The axis scales are clearly visible. Include numbers along axes only for bottom left plot of group (a 'group' is an analysis of identical markers).
- [ ] All plots are contour plots with outliers or pseudocolor plots.
- [ ] A numerical value for number of cells or percentage (with statistics) is provided.

Methodology

Sample preparation	HEK293A cells were transfected using a mixture of polyethylenimine and a plasmid encoding FLAG-tagged US28 variants.
Instrument	CytoFLEX flow cytometer (Beckman Coulter)
Software	FlowJo (BD Biosciences) and Prism 10 (GraphPad)
Cell population abundance	Not applicable, as no cell sorting was performed in this study.

Gating strategy	The main cell population was identified and gated based on forward scatter (FSC) and side scatter (SSC) to exclude cellular debris. Mean fluorescence intensity (MFI) of this primary population (~20,000 cells) was used for the analysis.

☐ Tick this box to confirm that a figure exemplifying the gating strategy is provided in the Supplementary Information.
