# Supplementary material for: Structural basis of stepwise G protein activation by the viral chemokine receptor US28
Source: Nat Commun. 2026 Sep 28;17:11171. doi: 10.1038/s41467-026-78122-7 (PMC13714248; doi:10.1038/s41467-026-78122-7)
Supplement: Supplementary file 3 [file 41467_2026_78122_MOESM3_ESM.pdf]

nature portfolio

Peer Review File

Structural basis of stepwise G protein activation by the viral chemokine receptor US28

Corresponding Author: Dr Naotaka Tsutsumi

This file contains all reviewer reports in order by version, followed by all author rebuttals in order by version.

Version 0:

Reviewer comments:

Reviewer #1

(Remarks to the Author)
Jude et al. describe a series of cryo-EM reconstructions and supporting biochemical studies that suggest they have snapshots of a series of conformational states along the trajectory of GDP release from Galphaq induced by a constitutively-active viral GPCR (US28). A large number of particles and exceptionally high resolution enable them to resolve from the same sample these substates, including one that resembles an encounter complex, which they call “transition-like” state (this was previously reported at lower resolution), an intermediate state (T2C) and then a nucleotide free state (C state). Even the non-enhanced maps are of reasonable high quality and there is little doubt about the presence or absence or placement of the GDP ligand. More important though is the conformation of the alpha5 helix of the G protein, which undergoes the most significant alterations among the structures, as one might expect from prior work on other class A GPCRs. We are both enthusiastic about the work.

Major comments we hope can be addressed:
1) It is interesting that under the same conditions one sees multiple stable sub-states. Is this a unique quality of US28 (if so, why might that be?) or is it something one might also expect for other GPCRs? Is it just that one hasn’t resolved these other structures at sufficiently high resolution? Or is it that they were using mini-Galpha’s or nb35 which mandates a nucleotide free complex, etc? Maybe the encounter (TL) complex is unique to this system given it involves noncanonical interactions between the receptor and the heterotrimer.
2) What evidence supports the TL complex being a “transition-like state”, and not T2C, which looks to be closer to the transition state. We appreciate that this is a semantic issue, but maybe a renaming of the states is in order for clarity.
3) We would like the authors to mention more caveats along the way, or in the discussion. For example, this is a micellar system, not a membrane, and they are using an Fab fragment that literally staples the two separable parts of the heterotrimer together, which could prevent them from undergoing physiological changes as they interact with receptor. Readers need to appreciate these nuances. We totally understand why it was necessary to manipulate the system using such tools to get these nice structures, but given that it wasn’t possible without the Fab it suggests that some of these observe substates may not reflect the whole story and that in reality the situation is much more dynamic.
4) The mutagenesis data where the authors knock out residues that are uniquely situated in each of the three substates is only consistent with the states being physiological, but it is not proof because loss of activity in these variants in cells could occur at any stage (e.g. from an allosteric effect or structural defect). In other words, one only knows that one loses activity, but one doesn’t know which state lost it, and most of the residues targeted could easily lead to structural defects given where they are situated in the complex. It would have been more convincing if the authors collected data on the mutants that they believe to be uniquely affecting one of the three substates and show that they lose that substate (or subsequent substates in their reaction coordinate) in their data. That’s a lot of work of course and is not being asked for by us. But it could be discussed.
5) We do not agree the authors’ conclusion that “the transition from initial engagement to the nucleotide-free state is gated by an allosteric checkpoint comprising the salt bridge between the conserved glutamate and arginine in the P-loop and Switch I”. Could it be that the loss of this salt bridge is simply just a consequence of changes in the guanine binding pocket as alpha5 engages the receptor cleft? Moreover, Alan Fersht has definitively shown that salt bridges at the surfaces of proteins do not stabilize them because each of the residues involved are equally happy in solvent than together (especially in presence of salt). To propose that this salt bridge is effectively the belt buckle that needs to burst to allow for GDP release seems a little absurd in this light. It seems more likely that the changes in this particular salt bridge are downstream of the changes at the N-terminus of alpha5 causing changes in the position of GDP. Couldn’t this shift instead be the “allosteric

switch"? Also, I don't see an alternative allosteric conduit that directly impacts the salt bridge that doesn't go through alpha5.

The short of it is, given all the uncertainties with the approaches used to get the structures, strong statements (such as "this is the allosteric switch") should be avoided and caveats given where appropriate.

6) Instead of the molecular dynamics simulations, which we don't find particularly compelling (a lot of caveats involved there as well) could the authors try heterogeneity analysis of their actual data (e.g. see https://www.nature.com/articles/s41467-025-67821-2)?

Minor comments/typos:

1) When reporting RMSD's one needs to specify how many atoms were in the calculation and what atoms were being compared. Also, given the resolution of the structures, one should be careful about how many significant figures are reported (e.g. 0.575 to 0.631 Å) should more realistically be reported as (0.58 to 0.63 Å). But to be honest ,given the resolution of the data, maybe (0.6 to 0.6 Å).
2) The buffer composition for the polishing SEC run of the complex should be specified. Seems important to evaluate the resulting conformational states and the potential influence of buffer components.
3) The kind of cells used for the functional/mutagenesis data should be specified.

Reviewer #2

(Remarks to the Author)
This study provides structural snapshots of the GPCR–G protein activation trajectory by capturing three distinct states of the US28–Gq complex. The identification of a putative intermediate state bridging GDP-bound and nucleotide-free conformations represents a potentially important advance in our understanding of G protein activation. The high quality of the resolved structures provides a solid structural basis for the authors' interpretations and conclusions. The manuscript is well written and clearly presented.
However, further clarification is required to establish the structural uniqueness and physiological relevance of this intermediate state. Several specific issues require further clarification prior to publication.
Major points
1. The authors propose a stepwise mechanism of G protein activation at US28. However, the structural snapshots alone are insufficient to establish a definitive temporal sequence. It would be helpful to perform real-time nucleotide exchange kinetics, time-resolved or single-molecular assays to directly support the proposed sequential activation model. Additional kinetic or dynamic evidence would substantially strengthen the conclusion that the observed states represent a true stepwise activation pathway rather than distinct conformational populations.
2. Although the manuscript discusses the differences between the intermediate state observed in US28 and that reported for μOR, a more comprehensive structural comparison would strengthen the analysis. In particular, a deeper evaluation of shared and distinct conformational features could further clarify the similarities and differences between viral and human GPCR activation mechanisms.
Minor points
1. In the Abstract, the TL-state, C-state, and T2C-state are introduced without definition. Please provide their full descriptions upon first mention to improve clarity for a broad readership.
2. In multiple instances, the Results section does not explicitly reference the corresponding figures, which may lead to confusion for readers. For example, in page 6 (lines 136–141) and page 8 (lines 190–197), structural findings are described without figure citations. The authors should carefully go through the manuscript to ensure that all descriptions are directly supported by referenced figures.
3. The manuscript would benefit from consistent amino acid nomenclature. For instance, residue 1293.50 is inconsistently denoted as "Arg1293.50" and "R1293.50" (page 6, lines 216, 219 and 221). Please standardize residue labeling throughout the text.
4. The contour levels used for visualization of the cryo-EM density maps in Fig. 2a are not indicated. In addition, GDP should be clearly labeled in the figure to improve readability.
5. Figure 4b presents comparisons between WT and mutant constructs; however, statistical significance is not indicated.
6. In Extended Data Fig. 7 (line 966), the manuscript refers to "four resolved states," which appears inconsistent with the three states (TL-state, T2C-state, and C-state) described in the main text. Please clarify this discrepancy.
7. In Extended Data Table 1, the ligand B factor for the T2C-state is reported as 4.31, which seems unusually low compared to typical cryo-EM values. In addition, the percentage of rotamer outliers (>3%) reported for some models is slightly higher than typically expected.

Reviewer #3

(Remarks to the Author)
Using cryo-EM, the authors report three different structures of a viral chemokine receptor US28 in complex with the G protein Gq, reconstituted in detergent micelle. The reported structures differ in the internal arrangement of the secondary structure elements of the G protein, orientation at the receptor interface and Gq-receptor interactions. The authors posit these structures as intermediates at various stages of G-protein activation. The manuscript provides some interesting observations regarding the dynamic changes in the G protein from initial recognition by the GPCR to final GDP expulsion. However, there are some major questions that remain to be addressed:

1. How physiologically relevant the intermediate T2C state is, given that the authors observed this state in detergent micelles, not a lipid bilayer? It is unclear whether the T2C state is an artifact of the artificial solvent environment, since transient states are less stable and therefore could be more affected by the change in solvent environment. The authors cite the MD simulations as justification in calling this an on-pathway intermediate, since 6/16 MD trajectories showed transition towards the C state. However, it supports but does not conclusively prove that the T2C state is a physiologically relevant intermediate. It is possible that there are multiple intermediate states, one of which is stabilized in detergent micelles and another in a lipid environment. Starting from any of these states, one might observe transition to the C state in MD simulations.

2. The mutagenesis section, as it is written, also doesn't sound entirely convincing in supporting the authors arguments regarding functional intermediates. It is necessary to clearly state the rationale for selecting each mutation target upfront by stating their hypothesis regarding each mutation's expected outcome and how that supports the existence of each intermediate state.

3. The authors give detailed description of the allosteric changes that occur within the G protein during various stages of activation. However, not many details are given on the allosteric changes within US28, since the receptor itself is a major component of the allosteric network that regulates Gq activation. Are there changes in major molecular switches within the receptor such as packing of hydrophobic/aromatic residues at the receptor core or major hydrogen bond networks, going from TL to T2C to the C state? Are there changes in the agonist binding site, interaction of the agonist with the receptor, or orientation of the EC and IC loops?

4. The authors claim extensive interactions between ICL2 of the receptor and the N helix of the G subunit. However, they have to be careful in interpreting such results since they used a chimeric Gq where the N helix is Gi related.

5. Methods section, lines 518-519. The choice of modeling some of the Glutamates and aspartates as neutral needs a clear rationale and supporting reference.

Version 1:

Reviewer comments:

Reviewer #1

(Remarks to the Author)
The authors have adequately addressed our major concerns.

Reviewer #2

(Remarks to the Author)
The authors have addressed all my concerns.

Reviewer #3

(Remarks to the Author)
The authors have responded constructively to my previous suggestions and concerns. There are some minor revisions to be made in the manuscript.
1. Physiological relevance of the T2C-state in a micellar environment
Response: The authors emphasize that the MD simulations were run in a POPC bilayer without scFv16, that the T2C-state was stably maintained over ~2 µs across 16 trajectories, and that transitions toward both the C- and E-states were observed. They add a "Conclusion and limitations of the study" section (lines 403–417), soften the claim to "plausible on-pathway intermediate" throughout, and cite the analogous "Engaged state" of Khan et al. in µOR-Gi.
Assessment: Adequate. The stability argument is correct, and the new limitations section makes the caveat clear to the readers.
A few minor refinements remain : (i) where the manuscript describes the T2C-state as capable of bidirectional transitions, the authors could report the trajectory counts explicitly, since movement toward the C-state was observed in 6/16 simulations whereas only 1/16 showed a partial reverse transition toward the E-state. (ii) the authors need to acknowledge that initiating all simulations from the T2C-state tests its stability and connectivity but does not survey alternative intermediates in the bilayer, with the C-state described in the Supplementary Text serving as a useful cross-reference. Finally, the caption for Supplementary Fig. 6c could be corrected to fix the wording error "away from the membrane and toward from GDP."
2. Rationale for mutation selection
Response: Lines 219–222 now state the three hypothesis classes upfront: persistent anchors, state-dependent contacts, and allosteric stabilization. Each mutant is introduced with its structural rationale. New Fig. 4c provides a focused contact diagram across the three states, and a caveat about endpoint assays is added at lines 266–268.
Assessment: Substantially improved. A minor revision is needed.
Remove residual deterministic phrasing. Lines 254–256 still state that the K2978.49A mutation "inhibits the E-to-T2C transition." However, an endpoint NanoBiT readout cannot support this mechanistic claim, and the wording is inconsistent with the caveat added twelve lines later. Please rephrase the statement as "is consistent with destabilization of the T2C- and C-states." I would also suggest adding, as an optional item, a compact supplementary table summarizing each mutant, the

state-dependence of its contact, the predicted outcome, and the observed LogRAi. This would allow readers to assess the predictive value of the framework directly and reduce the risk that the rationale appears post hoc.
3. Allosteric changes within US28 itself
Response: The letter states that US28 maintains a fully active 7TM conformation across the E-, T2C-, and C-states (Cα RMSD ~0.6 Å), that no macroscopic rearrangement was detected at the agonist-binding site, hydrophobic core packing, or the conventional molecular switches, and that US28 therefore acts as a rigid catalytic scaffold.
Assessment: This is a clear and interesting answer, and it does not appear in the revised text. The only related content is the global RMSD statement at lines 117–120 and a passing clause at line 271. A reader still cannot learn whether the receptor changes at all between states, which was precisely our question, since the receptor is a major component of the allosteric network.
This point requires only a minor revision: the authors could add a concise statement to the Results or Discussion explaining that US28 remains a rigid catalytic scaffold across the three states, supported by an appropriate supplementary comparison and framed with the relevant resolution caveat. This addition is important because the current manuscript does not yet make clear whether the receptor itself undergoes meaningful state-dependent structural changes, despite its central role in the proposed allosteric mechanism.
4. Chimeric Gq and interpretation of the ICL2–αN interface
Response: The limitations section (lines 409–411) now states that the Gαi1 substitution is confined to the first 18 N-terminal residues, spatially distant from the receptor interface, and that all Gαq residues contacting US28-ICL2 are native. Author Response Fig. 1 illustrates this.
Assessment: The concern is answered.
5. Neutral Glu/Asp residues in the simulations
Response: Lines 617–622 now give the rationale — Glu1243.45 and Glu1915.41 face the lipid membrane where a negative charge is unfavorable; Asp792.50 and Asp1283.49 are buried and evidenced to protonate upon activation — with citations to Ghanouni et al. (2000) and Ranganathan et al. (2014).
Assessment: Resolved for the buried residues; the citations are apt.
Minor revision. Asp1283.49 is modeled as neutral in the simulations, but the structural analysis describes it as forming an anion-quadrupole interaction with Tyr(-4) of the Gαq C-terminus (Supplementary Text, line 240), which implies that Asp1283.49 is charged in the static structure. Because D128A is also included as a mutagenesis target, the authors could add a sentence clarifying that the MD protonation state was chosen to model the activated receptor and does not affect the interpretation of the static structural analysis.

Open Access This Peer Review File is licensed under a Creative Commons Attribution 4.0 International License, which permits use, sharing, adaptation, distribution and reproduction in any medium or format, as long as you give appropriate credit to the original author(s) and the source, provide a link to the Creative Commons license, and indicate if changes were made.
In cases where reviewers are anonymous, credit should be given to 'Anonymous Referee' and the source.
The images or other third party material in this Peer Review File are included in the article's Creative Commons license, unless indicated otherwise in a credit line to the material. If material is not included in the article's Creative Commons license and your intended use is not permitted by statutory regulation or exceeds the permitted use, you will need to obtain permission directly from the copyright holder.
To view a copy of this license, visit https://creativecommons.org/licenses/by/4.0/

Reviewer #1:

Comments:

Jude et al. describe a series of cryo-EM reconstructions and supporting biochemical studies that suggest they have snapshots of a series of conformational states along the trajectory of GDP release from Galphaq induced by a constitutively-active viral GPCR (US28). A large number of particles and exceptionally high resolution enable them to resolve from the same sample these substates, including one that resembles an encounter complex, which they call "transition-like" state (this was previously reported at lower resolution), an intermediate state (T2C) and then a nucleotide free state (C state). Even the non-enhanced maps are of reasonable high quality and there is little doubt about the presence or absence or placement of the GDP ligand. More important though is the conformation of the alpha5 helix of the G protein, which undergoes the most significant alterations among the structures, as one might expect from prior work on other class A GPCRs. We are both enthusiastic about the work.

We sincerely thank the reviewer for the enthusiastic support and highly insightful comments, which have significantly improved the clarity of our manuscript. Please note that we have renamed the "transition-like" (TL) state to the "E-state" (encounter state) throughout the revised manuscript.

Major points:

1) It is interesting that under the same conditions one sees multiple stable sub-states. Is this a unique quality of US28 (if so, why might that be?) or is it something one might also expect for other GPCRs? Is it just that one hasn't resolved these other structures at sufficiently high resolution? Or is it that they were using mini-Galpha's or nb35 which mandates a nucleotide free complex, etc? Maybe the encounter (TL) complex is unique to this system given it involves noncanonical interactions between the receptor and the heterotrimer.

We thank the reviewer for this insightful point. We believe that these intermediate states were more readily trapped in our US28 system primarily because US28 possesses a short ICL2, which likely provides a weaker activation signal to the G protein from this specific interface compared to other class A GPCRs. We also agree that in many recent structural studies of GPCR-G protein complexes, the use of stabilizing tools (such as mini-G proteins, Nb35, dominant-negative Gα subunits, or specific tethering systems) along with nucleotide removal protocols biases the equilibrium toward the fully nucleotide-free state, likely precluding the capture of these transient intermediates. Therefore, we suspect that these pre-C-states are not entirely unique to US28, but rather exist in other GPCR systems as well. For instance, our E-state shares conceptual and structural similarities with the "Latent state" recently reported by Khan *et al.* for the μOR (*Nature*, 2025). Furthermore, considering observations of "reversible" systems, such as the transient snapshots of the β2AR-G_s complex upon GTP binding (Papasergi-Scott *et al.*, *Nature*, 2024), we speculate that initial encounter conformations analogous to our E-state complex are commonly sampled across other GPCRs.

2) What evidence supports the TL complex being a "transition-like state", and not T2C, which looks to be closer to the transition state. We appreciate that this is a semantic issue, but maybe a renaming of the states is in order for clarity.

We agree with the reviewer's comment regarding the nomenclature. The reviewer is correct that the original "TL-state" (transition-like) designation is misleading, particularly because the

subsequent intermediate state appears to structurally resemble a more plausible transitional state toward the nucleotide-free C-state. To eliminate this ambiguity, we have adopted the reviewer's suggestion and renamed these states throughout the revised manuscript and figures as follows:

(1) Encounter state (E-state): formerly the TL-state, representing the initial GDP-bound encounter complex.

(2) Transition-to-Canonical state (T2C-state): formerly the TL-to-C state. We have redefined the acronym to stand for "Transition-to-C," representing the plausible intermediate structurally transitioning to the C-state.

(3) Canonical state (C-state): retaining its original designation for the nucleotide-free state.

We thank the reviewer for this excellent suggestion, which has improved the clarity and flow of our structural narrative.

3) We would like the authors to mention more caveats along the way, or in the discussion. For example, this is a micellar system, not a membrane, and they are using an Fab fragment that literally staples the two separable parts of the heterotrimer together, which could prevent them from undergoing physiological changes as they interact with receptor. Readers need to appreciate these nuances. We totally understand why it was necessary to manipulate the system using such tools to get these nice structures, but given that it wasn't possible without the Fab it suggests that some of these observe substates may not reflect the whole story and that in reality the situation is much more dynamic.

While we explicitly mentioned the use of a detergent micelle system and the scFv16 fiducial marker at the very beginning of the Results section in our original manuscript, we fully agree that the implications of these tools, such as their potential to constrain physiological dynamics, must be more clearly discussed. To address the reviewer's point without distracting from the main conclusions of the paper, we have revised the manuscript as follows:

(1) We have systematically toned down deterministic language throughout the text to present our findings as a structurally grounded model rather than definitive proof.

(2) We have consolidated the detailed methodological caveats into a new section at the end of the Discussion, titled "Conclusion and limitations of the study."

In this new section, we address the constraints the reviewer raised: the detergent micelle environment, the chimeric Gα_q construct, and the stapling effect of scFv16. Importantly, we balance these caveats by noting that our MD simulations (performed in a lipid bilayer without scFv16) support the physical viability of the T2C-state, indicating that it is not merely an experimental artifact.

4) The mutagenesis data where the authors knock out residues that are uniquely situated in each of the three substates is only consistent with the states being physiological, but it is not proof because loss of activity in these variants in cells could occur at any stage (e.g. from an allosteric effect or structural defect). In other words, one only knows that one loses activity, but one doesn't know which state lost it, and most of the residues targeted could easily lead to structural defects given where they are situated in the complex. It would have been more convincing if the authors collected data on the mutants that they believe to be uniquely affecting one of the three substates

and show that they lose that substate (or subsequent substates in their reaction coordinate) in their data. That's a lot of work of course and is not being asked for by us. But it could be discussed.

We completely agree that endpoint cellular assays measure only final functional loss and cannot pinpoint exactly which intermediate state was structurally compromised. Following the reviewer's advice, we have explicitly discussed this caveat and toned down our claims. In particular, we have added a new sentence at the end of the functional assessment section (Results) to acknowledge that such functional readouts cannot isolate individual intermediate steps. We also reiterated this point in the newly added "Conclusion and limitations of the study" section in the Discussion. Furthermore, we have removed definitive terms throughout the manuscript. We now state that our mutagenesis data are "consistent with" the proposed stepwise mechanism, directly adopting the reviewer's suggested phrasing.

5) We do not agree the authors' conclusion that "the transition from initial engagement to the nucleotide-free state is gated by an allosteric checkpoint comprising the salt bridge between the conserved glutamate and arginine in the P-loop and Switch I". Could it be that the loss of this salt bridge is simply just a consequence of changes in the guanine binding pocket as alpha5 engages the receptor cleft? Moreover, Alan Fersht has definitively shown that salt bridges at the surfaces of proteins do not stabilize them because each of the residues involved are equally happy in solvent than together (especially in presence of salt). To propose that this salt bridge is effectively the belt buckle that needs to burst to allow for GDP release seems a little absurd in this light. It seems more likely that the changes in this particular salt bridge are downstream of the changes at the N-terminus of alpha5 causing changes in the position of GDP. Couldn't this shift instead be the "allosteric switch"? Also, I don't see an alternative allosteric conduit that directly impacts the salt bridge that doesn't go through alpha5.

The short of it is, given all the uncertainties with the approaches used to get the structures, strong statements (such as "this is the allosteric switch") should be avoided and caveats given where appropriate.

We thank the reviewer for this in-depth perspective. We completely agree with the reviewer that the disruption of the Glu49-Arg183 salt bridge is likely a downstream consequence of α5 engagement and the subsequent repositioning of the GDP molecule, rather than an independent, primary gate.

Indeed, this aligns with the mechanistic sequence we sought to describe: the insertion of α5 physically repositions the GDP molecule, which in turn pushes and breaks the Glu49-Arg183 salt bridge.

Regarding the thermodynamic stabilization, we fully concur with Dr. Fersht's principles that a simple surface salt bridge does not provide net protein stabilization. However, we respectfully wish to clarify that the Glu49-Arg183 interaction is not merely a generic surface salt bridge; it is uniquely coupled to, and stabilized by, the bound GDP in the inactive and E-states. Furthermore, the disruption of this specific GDP-stabilized interaction is functionally critical, as previous MD simulations (Sun *et al.*, *eLife* 2018) have proposed it to be the rate-limiting step in spontaneous G_q activation.

We realize, however, that our repeated use of phrases such as "gated by" and "allosteric checkpoint" gave the unintended impression that we viewed this salt bridge as the initial master switch. We agree that the use of such strong statements without proper context should be avoided given the methodological uncertainties.

Accordingly, we have carefully toned down these descriptions and removed overly dramatic metaphors throughout the revised manuscript. We now explicitly define the "salt bridge relay" strictly as a downstream, sequential cascade initiated by α5 engagement (e.g., stating in the Results that "our structures illustrate it as a consequence of α5 engagement"). Furthermore, we have contextualized the Glu49-Arg183 disruption not as a primary trigger, but as a critical intermediate event within the broader allosteric conduit driven by the receptor.

6) Instead of the molecular dynamics simulations, which we don't find particularly compelling (a lot of caveats involved there as well) could the authors try heterogeneity analysis of their actual data (e.g. see https://www.nature.com/articles/s41467-025-67821-2)?

We thank the reviewer for the excellent suggestion. Prompted by the reviewer's comment, we performed 3D Variability Analysis (3DVA) using our entire dataset of ~2.4 million "monomeric" complex particles (Author Response Video 1, uploaded to Figshare).

While 3DVA captures the global conformational variance between the major states, it fails to cleanly resolve the continuous trajectory passing through the specific transient intermediate T2C-state. This limitation primarily arises from an extreme population imbalance. The dataset is overwhelmingly dominated by the structurally distinct major states: ~0.8 million particles (~33%) adopt an E-like state, and ~1.1 million particles (~46%) adopt a C-like state. In stark contrast, the intermediate T2C-state is a highly transient, minor subpopulation comprising only ~70k particles (~3%). These particles were initially buried within the predominant C-like pool and could only be isolated by meticulously sub-classifying a subset that retained a closed α-helical domain.

The conformational landscape is further complicated by other minor populations, such as a "C'-state" (which broadly resembles the T2C-state but lacks visible GDP density, with its α5 helix base near the β6-α5 loop adopting a C-like conformation). Because the T2C-state represents only ~3% of the dataset, its subtle conformational signatures are easily overshadowed by the dominant motions and massive variance of the highly populated states. Consequently, 3DVA could not cleanly extract the specific trajectory passing precisely through the T2C-state.

Given this inherent limitation, we concluded that extensive discrete 3D classification was practically the only viable method to isolate and reconstruct the T2C-state from the bulk data. Because we cannot extract a clear continuous pathway directly from the cryo-EM data alone, we employed MD simulations as an indispensable complementary approach to bridge the dynamic gaps between our experimentally captured structural states and evaluate their multi-state transitions.

Minor points:

1) When reporting RMSD's one needs to specify how many atoms were in the calculation and what atoms were being compared. Also, given the resolution of the structures, one should be careful about how many significant figures are reported (e.g. 0.575 to 0.631 Å) should more realistically be reported as (0.58 to 0.63 Å). But to be honest ,given the resolution of the data, maybe (0.6 to 0.6 Å).

We have noted the number of Cα atoms compared in the transmembrane helices (line 114 in the original manuscript) and in the G_q alpha helical domains (line 264 in the original manuscript). Furthermore, following the advice, we have adjusted the significant figures for the reported RMSD values to reflect the resolution limits of our structural data more appropriately.

2) The buffer composition for the polishing SEC run of the complex should be specified. Seems important to evaluate the resulting conformational states and the potential influence of buffer components.

We agree that specifying the buffer composition is important for evaluating the resulting conformational states. We apologize for the omission and have now added the exact buffer composition for the polishing SEC run to the revised Methods section.

3) The kind of cells used for the functional/mutagenesis data should be specified.

We apologize for the oversight of initially including this information only in the reporting summary. We have now updated the Methods section to explicitly state that HEK293A cells (Thermo Fisher Scientific) were used for the functional and mutagenesis assays.

Reviewer #2:

Comments:

This study provides structural snapshots of the GPCR–G protein activation trajectory by capturing three distinct states of the US28–Gq complex. The identification of a putative intermediate state bridging GDP-bound and nucleotide-free conformations represents a potentially important advance in our understanding of G protein activation. The high quality of the resolved structures provides a solid structural basis for the authors' interpretations and conclusions. The manuscript is well written and clearly presented.

However, further clarification is required to establish the structural uniqueness and physiological relevance of this intermediate state. Several specific issues require further clarification prior to publication.

We are grateful to the reviewer for the positive evaluation and constructive feedback, which helped us better contextualize our structural findings.

Major points:

1. The authors propose a stepwise mechanism of G protein activation at US28. However, the structural snapshots alone are insufficient to establish a definitive temporal sequence. It would be helpful to perform real-time nucleotide exchange kinetics, time-resolved or single-molecular assays to directly support the proposed sequential activation model. Additional kinetic or dynamic evidence would substantially strengthen the conclusion that the observed states represent a true stepwise activation pathway rather than distinct conformational populations.

We agree with the reviewer that dynamic and kinetic evidence is ideally required to definitively prove the temporal sequence of the activation pathway. However, performing real-time kinetics or single-molecule assays for this specific complex is technically challenging. Such functional assays inherently require the biochemical isolation of a conformationally pure sample to serve as a synchronized starting point, yet our extensive cryo-EM 3D classification revealed that the purified sample is a highly heterogeneous mixture capturing multiple transient states.

Crucially, the intermediate states represent only a small fraction of this overall population. Out of our initial ~2.4 million particles, the E-like (referred to as TL-like in the previous version; ~33%) and C-like (~46%) states dominate, whereas the T2C-state comprises only ~3% (~70k particles), requiring focused 3D classification of a closed α-helical domain subset to identify. Furthermore, the mixture contains other minor populations, such as the GDP-lacking "C'-state" with a C-state-like α5 base (~3%, ~70k particles).

Because the structural differences between the T2C- and C'-states are subtle, distinguishing them is highly difficult without high-resolution structural data. Consequently, biochemically isolating a pure single-population sample of any of these states is exceedingly difficult. Furthermore, in conventional ensemble-averaged assays, the specific signal of the ~3% T2C intermediate would not only be indistinguishable from the other minor state, but it would also be completely masked by the dominant signals of the E- and C-states.

Given these fundamental experimental limitations, molecular dynamics (MD) simulations in a lipid bilayer represent the most viable methodology to interrogate this state. While experimental kinetic assays remain out of reach, our MD simulations are consistent with the sequential nature of these states. Crucially, trajectories starting from the T2C-state exhibited bidirectional

transitions: "forward" toward the C-state and "backward" toward the E-state. This provides compelling computational evidence that the T2C-state lies on a pathway bridging the E- and C-states.

2. Although the manuscript discusses the differences between the intermediate state observed in US28 and that reported for μOR, a more comprehensive structural comparison would strengthen the analysis. In particular, a deeper evaluation of shared and distinct conformational features could further clarify the similarities and differences between viral and human GPCR activation mechanisms.

We thank the reviewer for this excellent suggestion. We have added a new figure (Fig. 7) to provide a structural comparison between our intermediate states and those of the μOR-G_i complex (Latent vs. E-state; Engaged vs. T2C-state). Furthermore, we have expanded the Discussion section to evaluate potential reasons for the subtle differences observed, such as intrinsic family-specific dynamics and the distinct experimental methodologies utilized (i.e., our GDP removal protocol vs. the re-bound GDPβS protocol).

Minor points:

1. In the Abstract, the TL-state, C-state, and T2C-state are introduced without definition. Please provide their full descriptions upon first mention to improve clarity for a broad readership.

We thank the reviewer for this helpful suggestion. We have revised the Abstract to provide the full descriptions of these states. To maximize clarity and readability for a broad audience, we have decided to avoid using the abbreviations (such as E-state, T2C-state, and C-state) entirely in the Abstract. Additionally, as discussed in our response to Reviewer #1 Major Point 2, we have updated the nomenclature, renaming the "TL-state" to the "Encounter-state."

2. In multiple instances, the Results section does not explicitly reference the corresponding figures, which may lead to confusion for readers. For example, in page 6 (lines 136–141) and page 8 (lines 190–197), structural findings are described without figure citations. The authors should carefully go through the manuscript to ensure that all descriptions are directly supported by referenced figures.

We apologize for the omission. We have carefully reviewed the entire manuscript and added explicit figure citations to ensure that all structural descriptions in the Results section are directly supported by the corresponding figures.

3. The manuscript would benefit from consistent amino acid nomenclature. For instance, residue 1293.50 is inconsistently denoted as "Arg1293.50" and "R1293.50" (page 6, lines 216, 219 and 221). Please standardize residue labeling throughout the text.

We apologize for the inconsistency. We have standardized the nomenclature in the main text to the 3-letter + BW number format (e.g., $Arg129^{3.50}$) for structural descriptions. However, to optimize space and readability, we have consistently used the 1-letter format across all figures, as well as for denoting specific plasmid mutants (e.g., $R129^{3.50}A$ or R129A) in the text.

4. The contour levels used for visualization of the cryo-EM density maps in Fig. 2a are not indicated. In addition, GDP should be clearly labeled in the figure to improve readability.

We have revised Fig. 2a to directly indicate the contour levels used for the visualization of the cryo-EM density maps. In addition, GDP has been clearly labeled in the same figure. We believe these changes improve the readability.

5. Figure 4b presents comparisons between WT and mutant constructs; however, statistical significance is not indicated.

We thank the reviewer for highlighting this important omission. We have revised Fig. 4b to include statistical significance indicators (asterisks: * $P < 0.05$, ** $P < 0.01$) for all variants compared to their expression-matched wild-type controls. For the V294A variant, which showed a non-significant qualitative trend toward decreased signaling, we explicitly displayed the exact P-value ($P = 0.0897$) in the graph. Furthermore, we have added a dedicated "Statistical analysis" subsection to the Methods to clearly detail the specific statistical tests applied (e.g., two-tailed one-sample t-test and two-tailed unpaired t-test with Welch's correction, utilizing unadjusted P-values for *a priori* planned comparisons).

6. In Extended Data Fig. 7 (line 966), the manuscript refers to "four resolved states," which appears inconsistent with the three states (TL-state, T2C-state, and C-state) described in the main text. Please clarify this discrepancy.

We thank the reviewer for catching this typographical error. It was a remnant from an earlier draft. We have corrected the legend for Supplementary Fig. 7 (referred to as Extended Data Fig. 7 in the previous submission) to accurately refer to the "three resolved states," ensuring consistency.

7. In Extended Data Table 1, the ligand B factor for the T2C-state is reported as 4.31, which seems unusually low compared to typical cryo-EM values. In addition, the percentage of rotamer outliers (>3%) reported for some models is slightly higher than typically expected.

We sincerely thank the reviewer for carefully evaluating our models and catching these anomalies. Upon re-evaluating our initial modeling, we realized that the unusually low ligand *B*-factor was an artifact caused by map over-sharpening during post-processing. Prompted by the reviewer's comment, we resharpened the map for the T2C-state using a more conservative *B*-factor (-40 $Å^2$) and subsequently re-refined the model. This resolved the major anomalies: the GDP ligand *B*-factor is now appropriately refined to a more realistic 59.60 $Å^2$, and the overall model geometry has also improved, reducing the rotamer outliers to <3% for the key T2C-state.

Regarding the other models, we also reviewed their geometries. We found that the slightly elevated rotamer outliers in these remaining structures primarily correspond to side chains located in intrinsically more flexible regions with lower local resolution. For these dynamic areas, we opted not to enforce ideal rotamer geometries to avoid overfitting the limited density. We believe this approach provides the most faithful structural representation of the available data. We have updated the refinement statistics in Supplementary Table 1 (previously Extended Data Table 1) and regenerated the corresponding structural figures to reflect the improved T2C-state model.

Reviewer #3:

Comments:

Using cryo-EM, the authors report three different structures of a viral chemokine receptor US28 in complex with the G protein Gq, reconstituted in detergent micelle. The reported structures differ in the internal arrangement of the secondary structure elements of the G protein, orientation at the receptor interface and Gq-receptor interactions. The authors posit these structures as intermediates at various stages of G-protein activation. The manuscript provides some interesting observations regarding the dynamic changes in the G protein from initial recognition by the GPCR to final GDP expulsion. However, there are some major questions that remain to be addressed:

We appreciate the reviewer's critical reading and valuable suggestions, which have prompted us to refine our analyses and strengthen our conclusions.

1. How physiologically relevant the intermediate T2C state is, given that the authors observed this state in detergent micelles, not a lipid bilayer? It is unclear whether the T2C state is an artifact of the artificial solvent environment, since transient states are less stable and therefore could be more affected by the change in solvent environment. The authors cite the MD simulations as justification in calling this an on-pathway intermediate, since 6/16 MD trajectories showed transition towards the C state. However, it supports but does not conclusively prove that the T2C state is a physiologically relevant intermediate. It is possible that there are multiple intermediate states, one of which is stabilized in detergent micelles and another in a lipid environment. Starting from any of these states, one might observe transition to the C state in MD simulations.

We thank the reviewer for raising this important point. We fully agree with the inherent caveats of using detergent micelles for structure determination, although US28 and other GPCRs are known to facilitate GTP turnover in a micellar environment (Hilger *et al.*, *Science*, 2020; Tsutsumi *et al.*, *Sci Adv.*, 2022).

As noted by the reviewer, our MD simulations were performed in a lipid bilayer environment rather than in micelles. Importantly, in these simulations, we observed transitions not only toward the C-state but also toward the E-state. This bidirectional behavior strongly supports the T2C-state as a genuine on-pathway intermediate bridging the E- and C-states in a lipid bilayer.

We acknowledge, as currently discussed in the manuscript (newly added "Conclusion and limitations of the study" section), that we cannot exclude the existence of alternative pathways. The specific trajectories and energetics could be influenced by different lipid compositions and various other cellular factors (e.g., other proteins or ion gradients). However, the simulated complexes did not immediately transition away from the T2C-state; instead, this state was stably maintained over multiple simulations spanning several microseconds. If the T2C-state were merely an unstable artifact induced by the micellar environment, it is highly unlikely that it would exhibit such robust stability when embedded in a lipid bilayer. This observation strongly suggests that the T2C-state represents a physically viable conformation rather than a micelle-induced byproduct.

Additionally, a recent study by Khan et al. (*Nature*, 2025) captured a structurally analogous "Engaged state" during μOR-G_i activation. While this μOR structure was also solved in a detergent micelle, the independent observation of this intermediate across different GPCR and G protein families supports the concept that it represents a conserved functional milestone. Nevertheless, we have toned down our conclusions to describe the T2C-state as a "plausible on-pathway

intermediate" and explicitly addressed the caveats of the micellar environment in the newly added "Conclusion and limitations of the study" section in the Discussion.

2. The mutagenesis section, as it is written, also doesn't sound entirely convincing in supporting the authors arguments regarding functional intermediates. It is necessary to clearly state the rationale for selecting each mutation target upfront by stating their hypothesis regarding each mutation's expected outcome and how that supports the existence of each intermediate state.

We completely agree with this constructive feedback. We have revised the Results section to explicitly state the structural hypothesis and rationale for selecting each mutation target upfront (e.g., distinguishing between persistent anchors and state-dependent contacts). To help readers better visualize the state-dependent changes in interfacial contacts, we have added a new figure (Fig. 4c). This figure provides a contact map specifically focusing on the mutated US28 residues across the different states, while the comprehensive contact map for the entire interface is provided in Supplementary Fig. 7. Furthermore, as mentioned above, we have toned down our claims. We now explicitly state that our functional data are "consistent with" the proposed structural states, clearly acknowledging the inherent limitations of endpoint assays.

3. The authors give detailed description of the allosteric changes that occur within the G protein during various stages of activation. However, not many details are given on the allosteric changes within US28, since the receptor itself is a major component of the allosteric network that regulates Gq activation. Are there changes in major molecular switches within the receptor such as packing of hydrophobic/aromatic residues at the receptor core or major hydrogen bond networks, going from TL to T2C to the C state? Are there changes in the agonist binding site, interaction of the agonist with the receptor, or orientation of the EC and IC loops?

Our structural analysis indicates that US28 maintains a highly consistent, fully active 7TM conformation across the E-, T2C-, and C-states (Cα RMSD ~0.6 Å). Because the receptor is fully stabilized by the superagonist $CX_3CL1.44$ prior to nucleotide release, no noticeable macroscopic rearrangements were detected within the 7TM bundle during the E-to-C transition, specifically when analyzing the agonist-binding site, hydrophobic core packing, and conventional molecular switches. Acting as a rigid scaffold, this robust structural architecture enables US28 to function catalytically, facilitating the transient multi-state transitions of the coupling G protein.

4. The authors claim extensive interactions between ICL2 of the receptor and the αN helix of the Gα subunit. However, they have to be careful in interpreting such results since they used a chimeric Gq where the αN helix is Gi related.

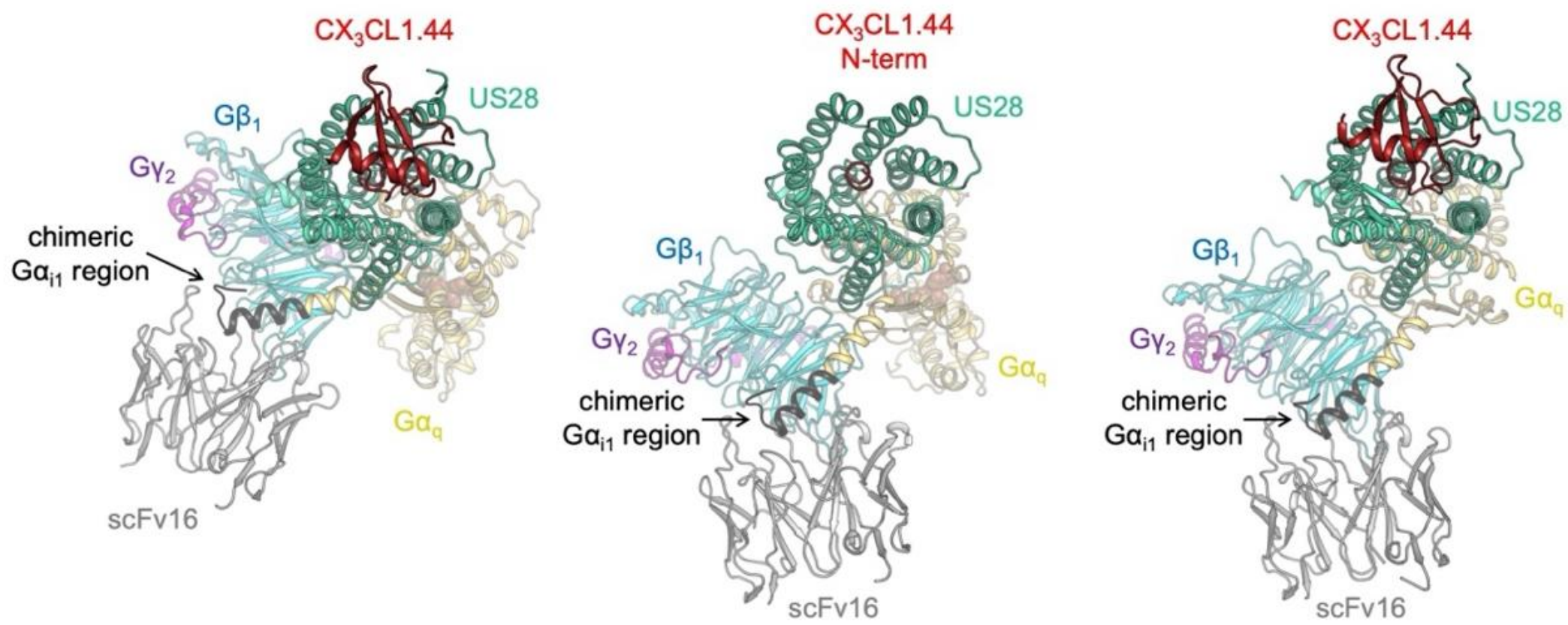

Author Response Fig. 1

We appreciate this cautionary note. We have clarified in the text that the chimeric $G\alpha_{i1}$ substitution is strictly limited to the first 18 N-terminal residues to enable scFv16 binding. As shown in Author Response Fig. 1, this region is spatially distant from the receptor interface, and all $G\alpha_q$ residues that interact with US28-ICL2 are native to G_q. Thus, while we acknowledge the inherent limitations of using a chimera and the stabilizing antibody, it is unlikely that the observed ICL2-αN interactions are artifacts of this approach.

5. Methods section, lines 518-519. The choice of modeling some of the Glutamates and aspartates as neutral needs a clear rationale and supporting reference.

We thank the reviewer for pointing this out. We have clarified these modeling choices. Briefly, two of the residues are in membrane-facing TM regions, where a negative charge would be highly energetically unfavorable. The other two are buried inside the receptor and are furthermore thought to be protonated in the active state of GPCRs (PMID: 10652295, PMID: 25347607).

Reviewer #3:

Comments:

The authors have responded constructively to my previous suggestions and concerns. There are some minor revisions to be made in the manuscript.

We sincerely thank the reviewer for the careful and constructive evaluation of our revised manuscript. We are pleased to hear that our previous responses and revisions have satisfactorily addressed your major concerns. Below, we provide our point-by-point responses to your remaining suggestions.

1. Physiological relevance of the T2C-state in a micellar environment

Response: The authors emphasize that the MD simulations were run in a POPC bilayer without scFv16, that the T2C-state was stably maintained over ~2 μs across 16 trajectories, and that transitions toward both the C- and E-states were observed. They add a "Conclusion and limitations of the study" section (lines 403–417), soften the claim to "plausible on-pathway intermediate" throughout, and cite the analogous "Engaged state" of Khan et al. in μOR-Gi.

Assessment: Adequate. The stability argument is correct, and the new limitations section makes the caveat clear to the readers.

A few minor refinements remain : (i) where the manuscript describes the T2C-state as capable of bidirectional transitions, the authors could report the trajectory counts explicitly, since movement toward the C-state was observed in 6/16 simulations whereas only 1/16 showed a partial reverse transition toward the E-state. (ii) the authors need to acknowledge that initiating all simulations from the T2C-state tests its stability and connectivity but does not survey alternative intermediates in the bilayer, with the C-state described in the Supplementary Text serving as a useful cross-reference. Finally, the caption for Supplementary Fig. 6c could be corrected to fix the wording error "away from the membrane and toward from GDP."

We thank the reviewer for the constructive suggestions.

First, while the exact trajectory counts were already detailed in the Results section, we agree that explicitly reporting them in the Discussion where the bidirectional transitions are summarized improves clarity. Accordingly, we have added the exact counts in parentheses to the relevant sentence.

Second, we fully agree with the your perspective regarding the limitation of our MD simulations. To address this, we have added a discussion acknowledging that initiating simulations exclusively from the T2C-state inherently limits the identification of other potential pathways, while noting that the experimentally resolved C'-state may offer a valuable structural reference for such alternative conformations.

Finally, we have corrected the typographical errors ("toward from" to "toward", and "an decrease" to "a decrease") in the legend for Supplementary Fig. 6c, as well as the manuscript throughout.

Please note that while the specific subheading "Conclusion and limitations of the study" was removed to comply with the journal's editorial formatting requests, the substantive content discussing these limitations is fully preserved as the final paragraph of the Discussion.

2. Rationale for mutation selection

Response: Lines 219–222 now state the three hypothesis classes upfront: persistent anchors, state-dependent contacts, and allosteric stabilization. Each mutant is introduced with its structural rationale. New Fig. 4c provides a focused contact diagram across the three states, and a caveat about endpoint assays is added at lines 266–268.

Assessment: Substantially improved. A minor revision is needed.

Remove residual deterministic phrasing. Lines 254–256 still state that the K2978.49A mutation "inhibits the E-to-T2C transition." However, an endpoint NanoBiT readout cannot support this mechanistic claim, and the wording is inconsistent with the caveat added twelve lines later. Please rephrase the statement as "is consistent with destabilization of the T2C- and C-states." I would also suggest adding, as an optional item, a compact supplementary table summarizing each mutant, the state-dependence of its contact, the predicted outcome, and the observed LogRAi. This would allow readers to assess the predictive value of the framework directly and reduce the risk that the rationale appears post hoc.

We agree with the reviewer that our previous wording regarding the $K297^{8.49}$A mutation was overly deterministic for an endpoint assay. We have revised the sentence to soften the mechanistic claim while retaining our proposed hypothesis as a possibility. It now states that the severe impairment of signaling by the $K297^{8.49}$A mutation is consistent with the destabilization of the T2C- and C-states, possibly by inhibiting the E-to-T2C transition.

Furthermore, we greatly appreciate the excellent suggestion to include a summary table. We have created a new Supplementary Table 4, which compactly summarizes each mutation target, the state-dependence of its contact, the structural hypothesis (predicted outcome), and the observed effect on signaling (LogRAi).

3. Allosteric changes within US28 itself

Response: The letter states that US28 maintains a fully active 7TM conformation across the E-, T2C-, and C-states (Cα RMSD ~0.6 Å), that no macroscopic rearrangement was detected at the agonist-binding site, hydrophobic core packing, or the conventional molecular switches, and that US28 therefore acts as a rigid catalytic scaffold.

Assessment: This is a clear and interesting answer, and it does not appear in the revised text. The only related content is the global RMSD statement at lines 117–120 and a passing clause at line 271. A reader still cannot learn whether the receptor changes at all between states, which was precisely our question, since the receptor is a major component of the allosteric network.

This point requires only a minor revision: the authors could add a concise statement to the Results or Discussion explaining that US28 remains a rigid catalytic scaffold across the three states, supported by an appropriate supplementary comparison and framed with the relevant resolution caveat. This addition is important because the current manuscript does not yet make clear whether the receptor itself undergoes meaningful state-dependent structural changes, despite its central role in the proposed allosteric mechanism.

We thank the reviewer for highlighting this omission. We fully agree that explicitly addressing the structural dynamics of the receptor in the text is essential for a comprehensive understanding of the allosteric network.

As suggested, we have incorporated a concise statement into the Results section explicitly characterizing fully activated US28 as a "rigid catalytic scaffold" during the progressive stages of G protein engagement. To visually support this conclusion, we have provided a new supplementary figure (Supplementary Fig. 5a; the original Supplementary Fig. 5a,b have been relabeled as Supplementary Fig. 5b,c), which aligns the US28 structures across the E-, T2C-, and C-states. To accommodate your point regarding the resolution caveat without disrupting the introductory flow of the main text, we have explicitly included this limitation in the corresponding figure legend.

4. Chimeric Gq and interpretation of the ICL2–αN interface

Response: The limitations section (lines 409–411) now states that the Gαi1 substitution is confined to the first 18 N-terminal residues, spatially distant from the receptor interface, and that all Gαq residues contacting US28-ICL2 are native. Author Response Fig. 1 illustrates this.

Assessment: The concern is answered.

We thank the reviewer for the positive assessment and are glad that our revisions have fully addressed this concern.

5. Neutral Glu/Asp residues in the simulations

Response: Lines 617–622 now give the rationale — Glu1243.45 and Glu1915.41 face the lipid membrane where a negative charge is unfavorable; Asp792.50 and Asp1283.49 are buried and evidenced to protonate upon activation — with citations to Ghanouni et al. (2000) and Ranganathan et al. (2014).

Assessment: Resolved for the buried residues; the citations are apt.

Minor revision. Asp1283.49 is modeled as neutral in the simulations, but the structural analysis describes it as forming an anion-quadrupole interaction with Tyr(-4) of the Gαq C-terminus (Supplementary Text, line 240), which implies that Asp1283.49 is charged in the static structure. Because D128A is also included as a mutagenesis target, the authors could add a sentence clarifying that the MD protonation state was chosen to model the activated receptor and does not affect the interpretation of the static structural analysis.

We appreciate the reviewer's careful reading and excellent suggestion to prevent any potential confusion for the readers. We completely agree that this methodological distinction between the dynamic simulation setup and the static structural interpretation should be explicitly stated.

To ensure the best consistency between our interpretation of the static structure and our MD simulation conditions, we have revised the description of the interaction in the Supplementary Text to read, "In the T2C- and C-states, however, the sidechain reorients such that its aromatic ring is sandwiched between US28-Cys66/Arg129 and is ultimately stabilized in the C-state by a thiol-π interaction, while the positive quadrupole of the ring edge is oriented toward the side chain carboxylic acid of US28-Asp128."
